# Life in an English Workhouse

**Published:** 1855-09

**Authors:** 


### LIFE IN AN ENGLISH WORKHOUSE.

An interesting paper on diet, read by Mr. Hunt before the Medical Society of London during the last session, and published in our last impression, gave rise at the time of its being read to an animated discussion regarding the internal economy of English workhouses. As many of our English readers may not be familiar with the proceedings of this Society, and as our foreign readers are possibly not well informed regarding the workhouse system in this country, we here offer a few remarks on the subject.

The modern workhouse in England is intended for the reception of those of the poor who are unable, either by youth, infirmity, or absolute poverty, to obtain the means of existence. The inmates consist, therefore, of old men and women, young children, and too often of able-bodied men and women out of work. Some of the sanitary arrangements in these institutions are excellent, and the mortality in them from acute diseases stands at a low figure. Other arrangements, however, are as highly defective, and lead to a very unnecessary amount of diseases which are not necessarily fatal. The selection of the site of the workhouse is in most cases judicious. The house is built outside the town to which it belongs, is surrounded by a pleasant and well-kept garden, and often presents an imposing appearance on entering the English town. The building arrangements are excellent. The wards, the passages and offices, are commodious, well-ventilated, and beautifully clean. The beds are comfortable and unencumbered by useless trappings, and the inmates are governed by a system which, though on some points absurdly rigorous, is on the whole conducive to order and cleanliness. These arrangements represent the fair side of the system, and account for the frequent immunity from those diseases of the epidemic or contagious class upon which so great a mortality in the world at large depends. In addition, the houses are regularly visited by competent medical men, who are invested with considerable powers in providing for the comfort of the subjects of disease.

With these facts before us, one would imagine *à priori* that diseases of all kinds should be nearly unknown in the English workhouse: there is pure air present, the occupants are in a partial quarantine, and medical skill is always at hand.

Unhappily, however, there remain some mistakes to prevent the attainment of that degree of health which might be anticipated. In visiting some of these institutions the mind is soon attracted by the miserable, desponding, unhealthy appearance of the occupants. These appearances are most common amongst the children. In one institution recently visited by us, this fact was painfully obvious. Scarcely a child presented a trace of the bloom and buoyancy of youth and health. They were all excitable, pale, weak, and dispirited; their features sharpened into an appearance of premature age. When spoken to, they shrank timidly, but sullenly, away, and many of them were strumous. They were, in short, all ill enough to be miserable and inactive, yet not sufficiently ill to be under medical care, nor did the governor and attendants seem to think that there was anything peculiar about them. We observed that throughout the whole establishment the same dejection and prostrated health prevailed. In this instance (the type of many others) the sanitary arrangements, of which we have spoken favourably were present, and were not a little dilated on by our dictatorial and hard-visaged guide, the major domo.

What, then, are the deeds of omission, or commission, which give rise to the unfavourable results above described? They are of two kinds, physical and moral. The main physical errors in the workhouse system relate to diet and exercise. The diet table, in every case that we are acquainted with, is conducted on a false principle, both as regards economy and health. The poor themselves generally conceive that they have not enough food supplied them. We believe they are to some extent correct in this supposition. But the main fact is, that the food they are supplied with does not, as food, represent the elements necessary for the development of an active vitality either of body or mind. From day to day, and from year to year, the same endless system too often prevails. Wretched stuff in the shape of gruel for breakfast, containing, as it is divided, scarcely a dash of nutritious matter ; animal food and vegetables two or three times a-week for dinner ; thin soup, bread and cheese or pudding on the intervening days, and a light bread supper at night, with tea for the aged, constitutes the diet role. The chemical student need not be told that such dietary can neither duly supply the fat which supports animal combustion, nor the staminal principles out of which blood, sinew, bone, and brain is made.

As regards exercise, great errors prevail. Confined to the wards and grounds of the institution, many of the elder and of the younger inmates scarcely know what it is to move the limbs freely, or inspire a chestful of air. This confinement begets a slothful inactivity, and depression of mind and body.

The moral errors are numerous. The education of the children, though good as far as it goes, is too limited and not sufficiently practical, while the mental energies of the elder inmates are frequently kept in unpardonable thraldom. Amusements there are none ; there are few books of an enlivening or instructive character ; nor are kind and cheering words from those in immediate authority too frequently spoken. We are not amongst those so-called philanthropists who would make a workhouse a paradise, and hold it out as an asylum for every lounging vagrant that bores us with his dismal complaints. The object of the institution, as we take it, ought to be to afford a home and a shelter for the orphan and indigent child, for the unfortunate mother, and the truly infirm poor. In this sense it is necessary, and might be turned not only to a charitable but to an useful account. The main evil attaching to the workhouse system now is, that it assumes too much of charity, and enforces too little of useful labour. Why should not the boys brought up in the workhouse be supplied with a mechanics' workshop, and be taught the use of the plane and the hammer ? Why should not the girls, in addition to the common routine of household duties, be taught some occupation which shall prevent them, when let loose on the world, from becoming mere kitchen drudges, or outcasts in society ? Why should not the able-bodied women, as Dr. Sieveking has proposed, be taught the duties of the sick room, and be educated in a scientific way for the office of the nurse, a functionary upon whose knowledge and intelligence the applications of medical skill rest so entirely? Why should not the days of the old man and woman be occasionally cheered with a gleam of sunshine and hope? And why should not all be fed on food which, according to the dictation both of science and instinct, best supplies the elements of the healthy body?

It should always be remembered, that the inmates of the English workhouse are, as a general rule, offenders against fortune rather than morality. Vast numbers of them are children, who are as innocent of crime as are the children of the great and the wealthy. On these unfortunates the utmost solicitude should be bestowed. The children of the poor constitute an important item in England's greatness. It remains for the public to decide whether, when they have these children under complete control, they will rear them into a healthy and generous manhood, or turn them adrift on the world rejoicing in a freedom for which they are not prepared, degenerate in body, and but little elevated in soul.

---

